# SEI-forming electrolyte additives for lithium-ion batteries: development and benchmarking of computational approaches

**DOI:** 10.1007/s00894-016-3180-0

**Published:** 2016-12-13

**Authors:** Piotr Jankowski, Władysław Wieczorek, Patrik Johansson

**Affiliations:** 1 Faculty of Chemistry, Warsaw University of Technology, ul. Noakowskiego 3, 00-664 Warsaw, Poland; 2 Department of Physics, Chalmers University of Technology, SE-412 96 Gothenburg, Sweden; 3 ALISTORE-ERI European Research Institute, 33 rue Saint Leu, 80039 Amiens, France; 4 Laboratoire de Réactivité et Chimie des Solides, CNRS UMR 7314, Université de Picardie Jules Verne, 33 rue Saint Leu, 80039 Amiens, France

**Keywords:** SEI-forming additive, Lithium-ion battery, DFT, Benchmark, Reduction

## Abstract

SEI-forming additives play an important role in lithium-ion batteries, and the key to improving battery functionality is to determine if, how, and when these additives are reduced. Here, we tested a number of computational approaches and methods to determine the best way to predict and describe the properties of the additives. A wide selection of factors were evaluated, including the influences of the solvent and lithium cation as well as the DFT functional and basis set used. An optimized computational methodology was employed to assess the usefulness of different descriptors.

**Electronic supplementary material:**

The online version of this article (doi:10.1007/s00894-016-3180-0) contains supplementary material, which is available to authorized users.

## Introduction

While high-capacity lithium-ion batteries (LIBs) have dominated the market for storage devices since the end of the twentieth century, there is still a growing need for better-performing batteries that can meet future energy demands [1–5]. The essential challenge is to develop a system with a much higher energy density than LIBs but which also at least matches LIBs in terms of cycle life. The stored energy is a product of the storage of charge in the electrodes (capacity) and the difference in potential between the electodes (voltage), and a popular way to improve the energy density is to increase the electrochemical potential difference between the cathode and anode. Since the potential of the commercially used anode, graphite, is already very close to the value for lithium metal (0.1 V vs. Li^+^/Li^0^) [6], the focus is on developing cathode materials that enable potentials as high as 5 V vs. Li^+^/Li^0^ to be feasible [7]. However, such a large potential difference poses a significant challenge in terms of selecting an appropriate electrolyte to use in the cell. The thermodynamic stability of the electrolyte is defined by its highest occupied molecular orbital (HOMO) and lowest unoccupied molecular orbital (LUMO) energies. Usually, the electrolyte is chosen to match the cathode potential and to provide kinetic stability at the anode–electrolyte interface. Currently applied electrolytes, based on mixtures of organic carbonates, undergo reduction processes at ca. 1 V vs. Li^+^/Li^0^ with the formation of a passivation layer—the solid electrolyte interphase (SEI). The SEI layer limits further reduction of the electrolyte and also affects many important battery parameters such as the capacity fade and power density. A spontaneously formed SEI causes a significant reduction in battery capacity, which is further worsened by subsequent charge–discharge cycles, so the application of a “functional” electrolyte is recommended [8]. Such an electrolyte consists of salt(s), solvent(s), and special functional additives; whereof SEI-forming additives are responsible for the controlled and rapid creation of an SEI layer.

SEI-forming additives have two main features: “high” reduction potentials and decomposition paths toward structures that can enable cation conduction between the anode and the electrolyte, such as oligo- or polymeric molecules similar to polymer electrolytes. There are a few groups of compounds that are known to fulfill these requirements. (i) Molecules containing unsaturated carbon–carbon bonds that provide an easy route for polymerization under reducing conditions. The most popular of these—vinyl carbonate (VC)—is able to form both types of polymeric species: poly(VC) and oligomers of VC [9–13]. Other compounds such as vinylethylcarbonate (VEC) [10, 14] and propargylmethyl carbonate (PMC) [15, 16] also effectively restrain the decrease in capacity. (ii) Molecules with halogen atoms such as fluorinated (FEC) [17] and chlorate carbonates (CEC) [18]. In these, the presence of the electronegative atom successfully initiates SEI creation, although the SEIs are usually more resistant and comprise various inorganic salts (LiF, LiCl). (iii) Sulfur-based compounds, mostly with structures similar to carbonates, such as organic sulfonates, sulfites, and sulfates. Ethylene sulfite (ES), the analog of EC, is very easily reduced at ca. 2 V vs. Li^+^/Li^0^, but the main product of its degradation is inorganic Li_2_SO_3_. Better properties may be obtained by using 1,3,2-dioxathiolane-2,2-dioxide (DTD), as the creation of a polymeric structure has been observed in this case [19, 20].

Computational studies are a popular means to develop better-performing additives, as they are faster and much less expensive than experimental trial-and-error testing. Many such studies have focused on explaining the reduction mechanisms associated with SEI formation, mainly for carbonate compounds such as EC, DMC, and VC [21–28], via a homolytic ring-opening mechanism. Less attention has been directed into the study of, for example, sulfur-containing compounds [29–31]. However, the most effective way to develop new additives appears to be computational screening [32–34], which involves focusing on specific features connected to a few descriptors. Such descriptors significantly simplify analysis when a very large number of compounds are considered. They enable the characteristics of new derivatives to be predicted based on fundamental molecular parameters. The most commonly used descriptors are the LUMO energy, the electron affinity (EA), and the chemical hardness (*η*). The first two describe the thermodynamic ability to accept a new electron and are used to assess the reduction potential, whereas *η* is a measure of reaction resistance and can serve as an indicator of the kinetics. Halls and Tasaki showed that a small *η* and low LUMO are favorable for SEI-forming additives [35]. Another couple of properties of importance were proposed by Park: the dipole moment (*μ*), and the binding energy with a lithium cation (BE). A higher *μ* leads to a stronger nonbonding interaction with Li^+^, whereas weak binding between the additive and the lithium cation facilitates processes on the anode and ensures rapid formation of the SEI [36]. In some cases, a sufficient change in bond length could also be a descriptor that predicts the vulnerability of a bond to the fragmentation of the additive during the reduction process [33].

To obtain trustworthy predictions for applications, it is crucial to apply a proper methodology, encompassing the selection of a method, model, and computational approach [37]. In 2004, Han tested several different DFT functionals with the aim of evaluating additive performance by describing the reduction of EC and VC [26]. However, since then there has been further progress in this area of research, including a new family of Minnesota functionals [38–41]. Several redox-process benchmarking papers have already been published which show that some functionals, especially M06-2X, provide better results than previously thought [42–45]. However, while they generally show improved performance as compared to wavefunction-based methods, it is essential to compare the results they yield to concrete experimental data for additives used in battery electrolytes in order to identify the best methodology. Another issue is how to include the effects of solvents: Lespes et al. [46] recommend the use of an implicit solvent model coupled with an explicit representation of the first solvation shell. However, as this approach is computational expensive, especially if there are a large number of molecules to be tested and their stable configurations are not known, implicit models are usually used. The type of solvent and its permeability (dielectric constant) applied in the model vary, as does the approach used to calculate the reduction potential (e.g., the effect of salt is the source of debate) [27–29, 47, 48].

**Fig. 1:**
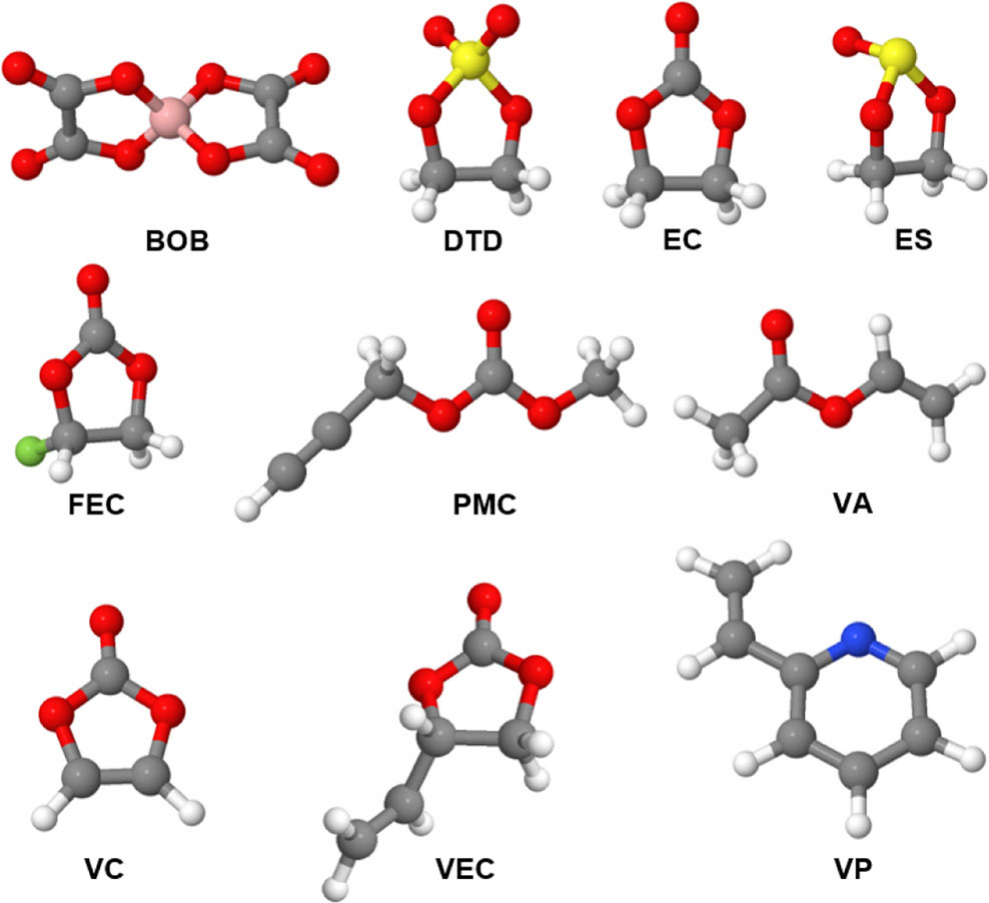
Chemical structures of the SEI-forming additives; all geometries were optimized using C-PCM M06-2X/6-311++G(d,p)

In the work reported in the present paper, we compared different ways of predicting reduction behavior using a methodological benchmark. Various basis sets, DFT functionals, and solvation models were tested. Finally, a methodology was chosen, and a search for appropriate descriptors was conducted. All of the data were generated for several well-known SEI-forming additives (EC, FEC, VC, VEC, PMC, vinyl acetate (VA) [16], 2-vinyl pyridine (VP) [49], ES, DTD, and BOB; Fig. 1), enabling us to correlate the chosen methodology with corresponding experimental data.

## Computational details

**Fig. 2a–c:**
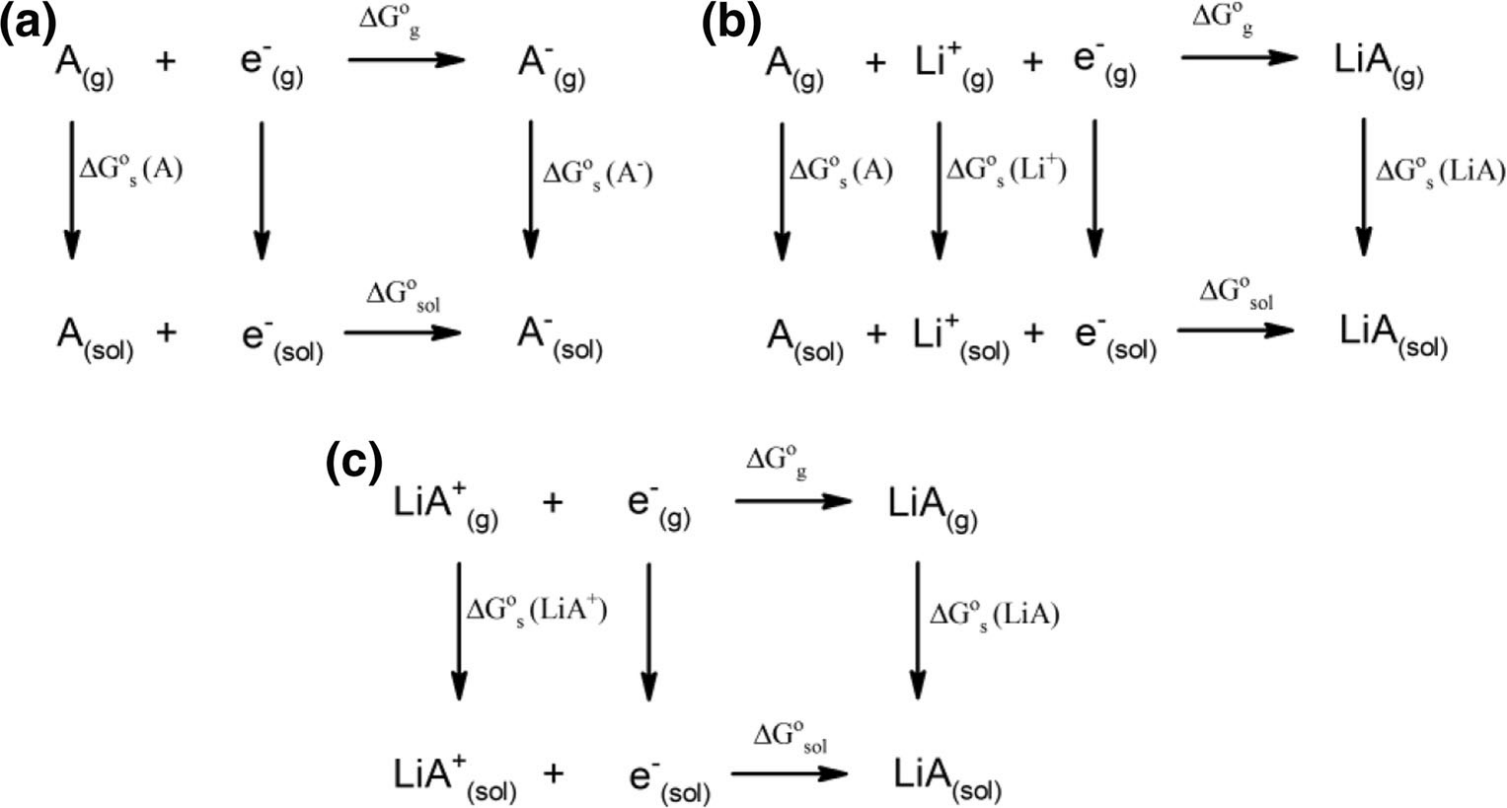
The three thermodynamic cycles—(**a**), (**b**), and (**c**)—used to calculate reduction potentials. As the change in energy of an electron upon switching from a vacuum to solution is very small, it was neglected (i.e., defined as exactly zero).* A* denotes the additive

All calculations were carried out with the Gaussian09 package [50] using Hartree–Fock (HF) and several DFT functionals: mPW2PLYP [51], TPSSh [52], B2PLYP [53], B3LYP [54], VSXC [55], PBE0 [56], M06-2X [39], M06L [57], M11 [40], and MN12L [41]. Four triple-zeta basis sets with polarization and diffuse functions from different families were tested: 6-311++G(d,p) [58, 59], def2-TVPD [60], aug-pcseg-2 [61], and aug-cc-pVTZ [62]. The influence of solvent was examined using the conductor-variant polarized continuum model (C-PCM) [63, 64] with the M06-2X functional. Tetrahydrofuran (*Ɛ*_r_ =7.4), acetone (*Ɛ*_r_ = 20.5), acetonitrile (*Ɛ*_r_ = 35.7), and water (*Ɛ*_r_ = 78.4) were employed as model solvents. All of the geometries were optimized both in vacuum and in each of the solvents tested. The reduction potentials were calculated in three different ways using thermodynamic cycles (Fig. 2): (a) ignoring the influence of the lithium cation, (b) assuming the simultaneous reduction and coordination of the lithium cation (electroneutrality); and (c) assuming that the lithium cation is coordinated to the molecule during the entire reduction process. A correction of −1.46 V was used to convert absolute potentials to the Li^+^/Li^0^ scale [65]. Additionally, several descriptors (*μ*, HOMO, LUMO, IP, EA, *η*, and BE) were determined for each additive, based on the optimized ground-state structure. A–Li^+^ ion-pair complexes were obtained by optimizing the starting geometries generated by the random insertion of Li^+^ at 25 different positions relative to each choice of A.

## Results and discussion

We performed our benchmarking in three steps: first, we tested the effects of varying the solvent model and the dielectric constant; second, for a chosen solvation mode, we carried out DFT and basis set benchmarking; finally, using the best-performing methodology, we searched for descriptors that could enhance the computational screening of SEI-forming additives.

### Influence of the solvent model

**Fig. 3a–d:**
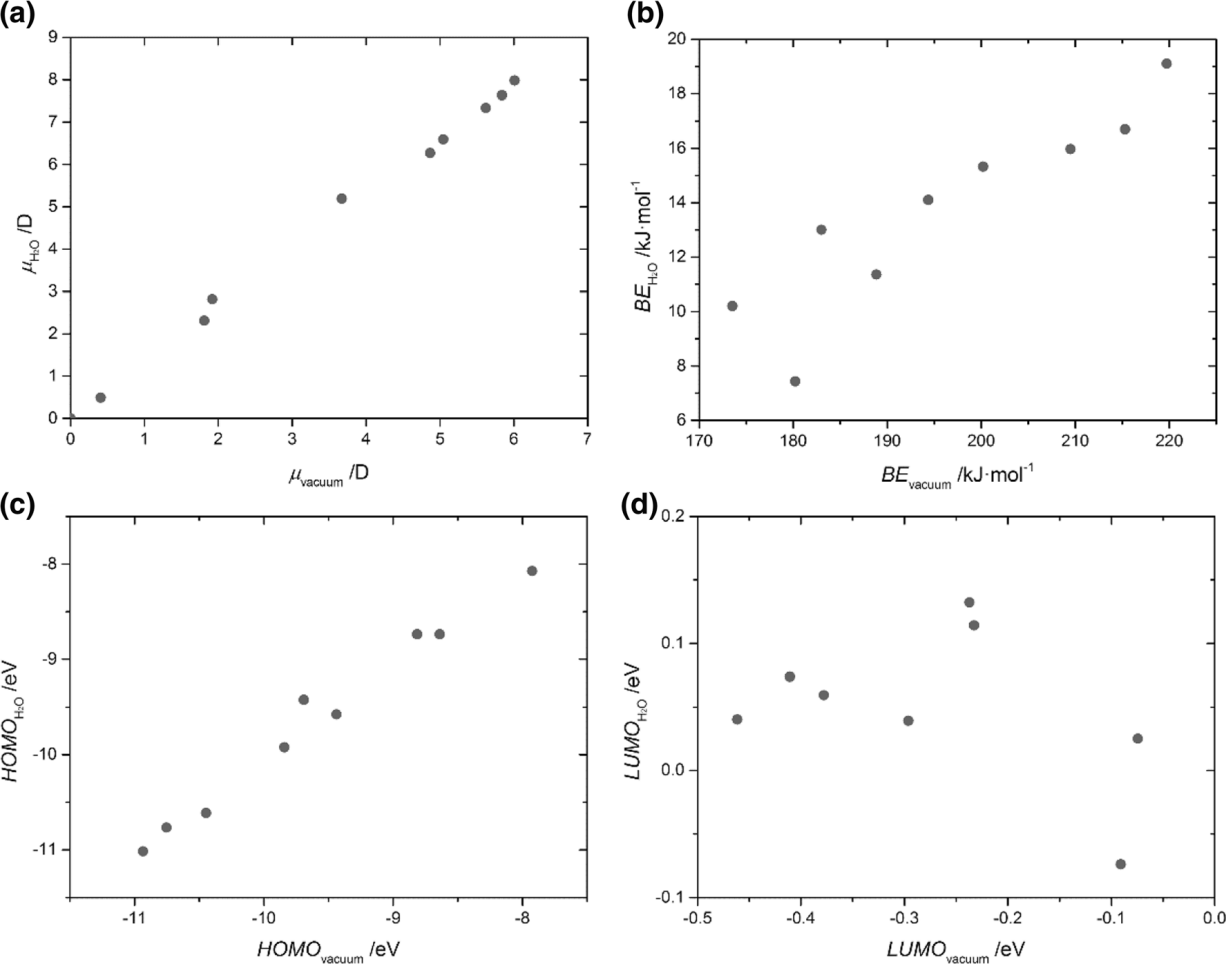
Comparison of dipole moments (**a**), BEs (**b**), HOMO energies (**c**), and LUMO energies (**d**) calculated in the gas phase and by C-PCM (in water)

Two well-known SEI-forming additives of LIBs, EC and DTD, were chosen for study, and selected molecular properties of those additives were analyzed for different dielectric constants using the C-PCM, and the results were compared with those from vacuum computations (Table 1). Upon shifting from vacuum to solution, larger dipole moments were observed (a result of the polarization of the solute by the solvent [66, 67]), and a linear relationship was seen (Fig. 3a). In addition, a small increase in the HOMO–LUMO gap was observed. This was mostly an effect of an increased LUMO rather than a decreased HOMO, as the latter is quite insensitive to solvation (Fig. 3c, d). Similar dependences were also noted for IP, EA, and *η*, as they have the same origin. A significant decrease was observed in the BE, which is logical given that solvents facilitate the dissociation of ion pairs: the BEs upon solvation in water were 4–8% of the BEs in vacuum (Fig. 3b). Since BOB is an anion, it behaved differently; its data points were out of range of the plots presented in Fig. 3.

**Table 1:** Comparison of the dipole moments, HOMO and LUMO energies, ionization potentials, electron affinities, chemical hardnesses, binding energies, and reduction potentials determined using different dielectric constants in C-PCM and M06-2X/6-311++G(d,p) calculations

		EC					DTD				
		Vacuum	THF	Acetone	ACN	Water	Vacuum	THF	Acetone	ACN	Water
		*ɛ*_r_ = 1	*ɛ*_r_ =7.4	*ɛ*_r_ =20.5	*ɛ*_r_ =35.7	*ɛ*_r_ =78.4	*ɛ*_r_ =1	*ɛ*_r_ =7.4	*ɛ*_r_ =20.5	*ɛ*_r_ =35.7	*ɛ*_r_ =78.4
*μ* (D)		5.62	7.06	7.25	7.30	7.33	6.01	7.66	7.88	7.94	7.98
HOMO (eV)		−10.45	−10.61	−10.62	−10.62	−10.62	−10.75	−10.77	−10.77	−10.77	−10.77
LUMO (eV)		−0.38	0.02	0.05	0.05	0.06	−0.46	0.00	0.03	0.04	0.04
IP (eV)		11.44	9.68	9.47	9.41	9.38	11.83	9.82	9.63	9.63	9.55
EA (eV)		−1.06	0.43	0.58	0.62	0.65	−0.48	0.36	0.55	0.61	0.65
*η* (eV)		6.25	4.63	4.44	4.40	4.36	6.15	4.73	4.54	4.51	4.45
BE (kJ mol^−1^)		209.5	38.64	22.29	18.64	15.97	180.22	26.11	12.23	9.57	7.43
*E*_red_ (V vs. Li^+^/Li^0^)	a	−1.11	0.97	1.18	1.23	1.27	−1.87	1.58	1.85	1.90	1.94
	b	5.85	2.15	1.78	1.70	1.64	6.34	2.70	2.34	2.31	2.19
	c	3.80	1.90	1.71	1.67	1.64	4.60	2.55	2.33	2.30	2.24
	Exp.	1.36 [69]					2.13 [19]

Considerable changes in predicted reduction potentials were observed due to the shifted LUMO levels and the influence of the solvent. Indeed, the directions of the changes depended on the approach applied. Neglecting the lithium cation (a) resulted in very low reduction potentials in the gas phase. Introducing Li^+^ into the system (b, c), even when it was only in present in its reduced form, increased the reduction potentials. When solvent effects were taken into account, the predicted reduction potentials moved even closer to the experimentally observed values, so this is necessary for any accurate quantitative prediction of *E*_red_. The results for different solvents (i.e., dielectric constants) show that varying the dielectric constant only had a significant impact at low values of this constant [68]; the effect of any increase in the constant was minimal when *Ɛ*_r_ was greater than approximately 20. Thus, water—the best-parameterized solvent—can be used as a decent approximation for all solvents with *Ɛ*_r_ > 20 [34], so water was used as the solvent in all subsequent calculations.

### DFT functional and basis-set benchmarking

**Fig. 4a–c:**
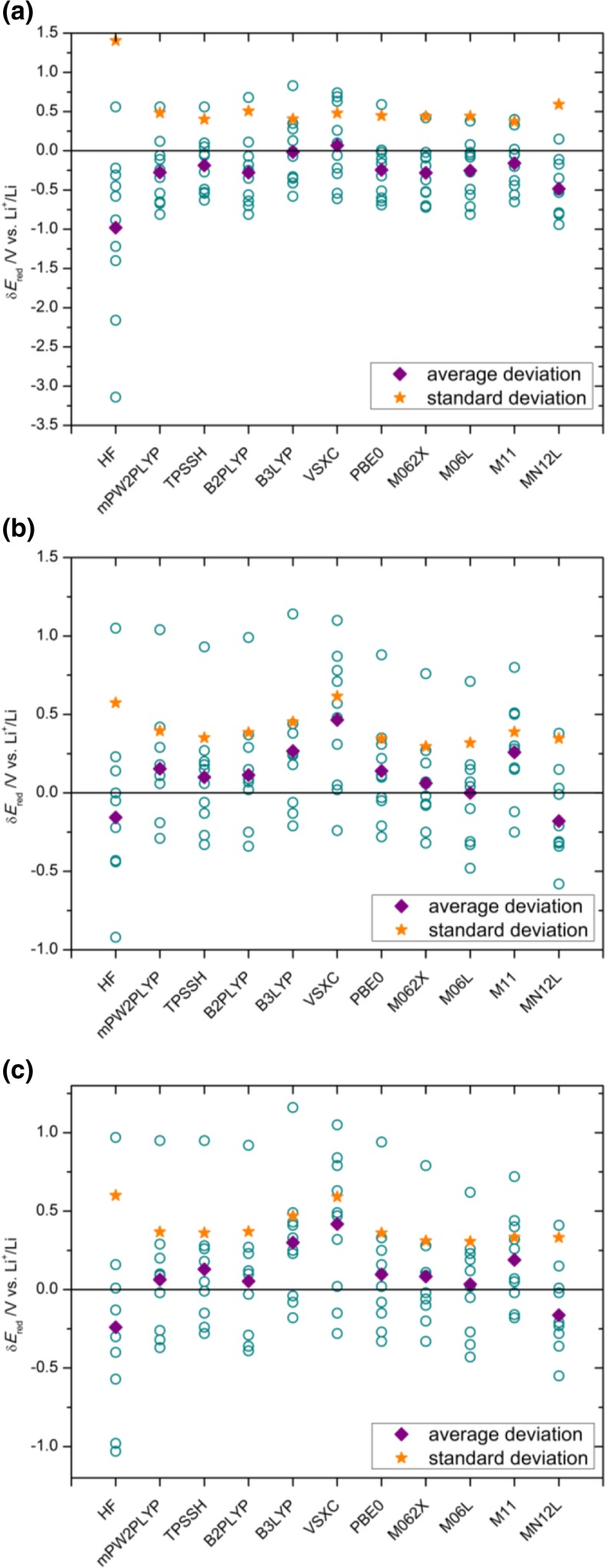
Deviation, δ*E*_red_, of the reduction potential predicted using thermodynamic cycle (a), (b), or (c) (plots** a–c**, respectively) and each DFT functional from the corresponding experimental value. Average and standard deviations are also shown

The reduction potential is the most important parameter that determines if and when a given compound can be used as an SEI-forming additive. Thus, we calibrated the computational method against experimental reduction potential data from electrochemical studies [16, 19, 28, 49, 69–72]. To determine the best DFT functional, a Pople basis set was used: 6-311++G(d,p). Analysis of the three thermodynamic cycles showed that the influence of the lithium cation often had to be considered (Table 2). Lower standard deviations were only obtained without Li^+^ (i.e., with thermodynamic cycle (a)) when the B3LYP and VSXC functionals were applied. For most methods, the difference between the results obtained using cycles (b) and (c) was very small. To further compare the different methods, the averages and standard deviations obtained using the computational method with various functionals were compared to the corresponding experimental data (Fig. 4). Overall, the M06-2X functional was found to be the best performer, although the other functionals of the Minnesota family (M06L, M11, and MN12L) also provided good results. This is consistent with other reported studies that have benchmarked the DFT prediction of redox properties against highly accurate wavefunction methods [42–45].

**Table 2:** Reduction potentials predicted using the three different thermodynamic cycles (a), (b), and (c) and various functionals; all values shown in the table are in V vs. Li^+^/Li^0^

Additive	Exp.	Thermodynamic cycle	HF	mPW2PLYP	TPSSH	B2PLYP	B3LYP	VSXC	PBE0	M062X	M06L	M11	MN12L
EC^a^	1.36 [69]	a	1.05	1.30	1.32	1.29	1.49	1.62	1.26	1.27	1.28	1.38	1.01
		b	1.50	1.78	1.63	1.73	1.80	2.07	1.71	1.63	1.43	1.86	1.35
		c	1.37	1.65	1.62	1.63	1.79	1.99	1.61	1.64	1.59	1.76	1.34
FEC^a^	0.7 [70]	a	0.48	0.82	0.80	0.81	1.05	1.44	0.71	0.68	0.78	1.03	0.59
		b	0.65	0.99	0.85	0.99	1.08	1.57	0.92	0.77	0.85	1.21	0.85
		c	0.57	0.90	0.88	0.93	1.11	1.54	0.86	0.79	0.89	1.14	0.71
VC^a^	1.40 [69]	a	0.82	0.86	0.87	0.84	1.07	1.11	0.80	0.87	0.91	0.96	0.61
		b	1.18	1.21	1.07	1.15	1.27	1.45	1.12	1.15	1.09	1.15	0.82
		c	1.10	1.14	1.12	1.11	1.32	1.42	1.07	1.20	1.13	1.22	0.85
VEC^a^	2.2 [28]	a	1.75	1.86	1.93	1.85	2.13	2.30	1.88	1.83	1.93	2.00	1.67
		b	2.20	2.31	2.26	2.27	2.45	2.77	2.31	2.18	2.24	2.46	1.99
		c	2.07	2.18	2.25	2.17	2.45	2.69	2.22	2.18	2.22	2.18	1.99
PMC^a^	0.83 [16]	a	1.39	1.39	1.39	1.51	1.66	1.52	1.42	1.25	1.21	1.23	0.98
		b	1.88	1.87	1.76	1.82	1.97	1.93	1.71	1.61	1.54	1.63	1.21
		c	1.80	1.78	1.78	1.75	1.99	1.88	1.77	1.64	1.45	1.55	1.24
VA	0.88 [16]	a	−1.28	0.07	0.25	0.07	0.30	0.27	0.19	0.16	0.07	0.23	−0.06
		b	−0.04	0.59	0.61	0.54	0.67	0.64	0.67	0.56	0.40	0.76	0.30
		c	−0.10	0.51	0.64	0.49	0.70	0.60	0.61	0.55	0.45	0.72	0.33
VP	0.8 [49]	a	−0.08	0.56	0.74	0.55	0.79	0.74	0.78	0.72	0.75	0.76	0.63
		b	0.36	0.91	1.00	0.89	1.04	1.11	1.11	0.99	0.98	1.10	0.83
		c	0.40	0.89	1.08	0.90	1.13	1.12	1.13	1.08	1.06	1.12	0.95
ES^a^	2.1 [71]	a	0.88	1.45	1.56	1.46	1.75	1.88	1.46	1.40	1.54	1.54	1.31
		b	1.67	2.16	2.04	2.12	2.28	2.58	2.07	2.03	2.00	2.25	1.79
		c	1.53	2.08	2.09	2.07	2.33	2.57	2.02	2.04	2.05	2.17	1.82
DTD^a^	2.13 [19]	a	−1.01	2.02	2.18	1.95	2.41	2.76	1.97	1.94	2.11	2.08	1.63
		b	2.36	2.31	2.31	2.28	2.57	2.91	2.23	2.19	2.20	2.41	1.81
		c	2.29	2.23	2.39	2.25	2.62	2.92	2.23	2.24	2.25	2.39	1.90
BOB	1.8 [72]	a	0.40	1.13	1.31	1.10	1.39	1.26	1.29	1.28	1.09	1.42	0.99
		b	0.88	1.61	1.67	1.55	1.74	1.82	1.75	1.72	1.47	1.96	1.46
		c	0.77	1.48	1.65	1.44	1.76	1.65	1.65	1.70	1.45	1.85	1.44
Standard deviation		a	1.41	0.48	0.40	0.51	0.41	0.48	0.45	0.44	0.44	0.37	0.59
		b	0.57	0.39	0.35	0.38	0.45	0.62	0.34	0.30	0.32	0.39	0.35
		c	0.60	0.37	0.36	0.37	0.47	0.59	0.36	0.31	0.31	0.33	0.33
Average deviation		a	−0.98	−0.27	−0.19	−0.28	−0.02	0.07	−0.24	−0.28	−0.25	−0.16	−0.48
		b	−0.16	0.15	0.10	0.11	0.27	0.47	0.14	0.06	0.00	0.26	−0.18
		c	−0.24	0.06	0.13	0.05	0.30	0.42	0.10	0.08	0.03	0.19	−0.16

^a^A chemical bond is broken during the reduction

Based on the Minnesota family of DFT functionals, the impact of the type of basis set used was examined using cycle (b) (Table 3). The standard deviations obtained with the various the basis sets considered confirmed that M06-2X was the best functional. The popular 6-311++G** basis set gave quite good results compared to the other larger and more advanced basis sets; only the Dunning basis set, aug-cc-pVTZ, provided data that were in better agreement with the experimental data. The Dunning basis set is, however, computationally much more expensive; 46 basis functions are used for each first-row atom, while all other basis sets comprise 22–24 basis functions. Hence, due to its combination of accuracy and efficiency, we chose the Pople basis set 6-311++G(d,p) for subsequent use.

**Table 3:** Reduction potentials obtained using thermodynamic cycle (b) and various functionals and basis sets; all values are in V vs. Li^+^/Li^0^

Additive	Exp.	Basis set	M06-2X	M06L	M11	MN12L
EC*	1.36	6-311++G**	1.63	1.43	1.86	1.35
		def2-TVPD	1.66	1.45	1.78	1.25
		aug-pcseg-2	1.68	1.44	1.80	1.30
		aug-cc-pVTZ	1.59	1.49	1.64	1.18
FEC*	0.7	6-311++G**	0.77	0.85	1.21	0.85
		def2-TVPD	0.72	0.68	1.00	0.62
		aug-pcseg-2	0.72	0.57	0.98	0.38
		aug-cc-pVTZ	0.72	0.72	1.02	0.63
VC*	1.40	6-311++G**	1.15	1.09	1.15	0.82
		def2-TVPD	1.09	0.91	1.19	0.65
		aug-pcseg-2	1.10	0.90	1.21	0.72
		aug-cc-pVTZ	1.06	0.96	1.08	0.59
VEC*	2.2	6-311++G**	2.18	2.24	2.46	1.99
		def2-TVPD	2.19	2.13	2.40	1.86
		aug-pcseg-2	2.22	2.12	2.43	1.92
		aug-cc-pVTZ	2.13	2.14	2.24	1.79
PMC*	0.83	6-311++G**	1.59	1.54	1.63	1.21
		def2-TVPD	1.39	1.21	1.49	1.28
		aug-pcseg-2	1.46	1.30	1.52	1.36
		aug-cc-pVTZ	1.49	1.30	1.44	1.36
VA	0.88	6-311++G**	0.56	0.40	0.76	0.30
		def2-TVPD	0.61	0.29	0.78	0.23
		aug-pcseg-2	0.31	0.15	0.37	0.21
		aug-cc-pVTZ	0.54	0.37	0.62	0.23
VP	0.8	6-311++G**	0.99	0.98	1.10	0.83
		def2-TVPD	1.04	0.89	1.13	0.81
		aug-pcseg-2	1.05	0.90	1.15	0.88
		aug-cc-pVTZ	0.94	0.84	0.94	0.69
ES*	2.1	6-311++G**	2.03	2.00	2.25	1.79
		def2-TVPD	1.65	1.50	1.82	1.20
		aug-pcseg-2	1.70	1.54	1.86	1.30
		aug-cc-pVTZ	1.80	1.75	1.91	1.44
DTD*	2.13	6-311++G**	2.19	2.20	2.41	1.81
		def2-TVPD	1.50	1.37	1.67	0.94
		aug-pcseg-2	1.62	1.46	1.73	1.09
		aug-cc-pVTZ	1.79	1.79	1.86	1.24
BOB	1.8	6-311++G**	1.72	1.47	1.96	1.46
		def2-TVPD	1.80	1.40	1.96	1.37
		aug-pcseg-2	1.72	1.31	1.83	1.30
		aug-cc-pVTZ	1.71	1.40	1.76	1.30
Standard deviation		6-311++G**	0.30	0.32	0.39	0.35
		def2-TVPD	0.35	0.42	0.35	0.60
		aug-pcseg-2	0.34	0.42	0.34	0.56
		aug-cc-pVTZ	0.28	0.34	0.30	0.49
Average deviation		6-311++G**	0.06	0.00	0.26	−0.18
		def2-TVPD	−0.05	−0.23	0.11	−0.39
		aug-pcseg-2	−0.03	−0.22	0.11	−0.36
		aug-cc-pVTZ	0.03	−0.14	0.11	−0.29

### Descriptor search

**Fig. 5a–b:**
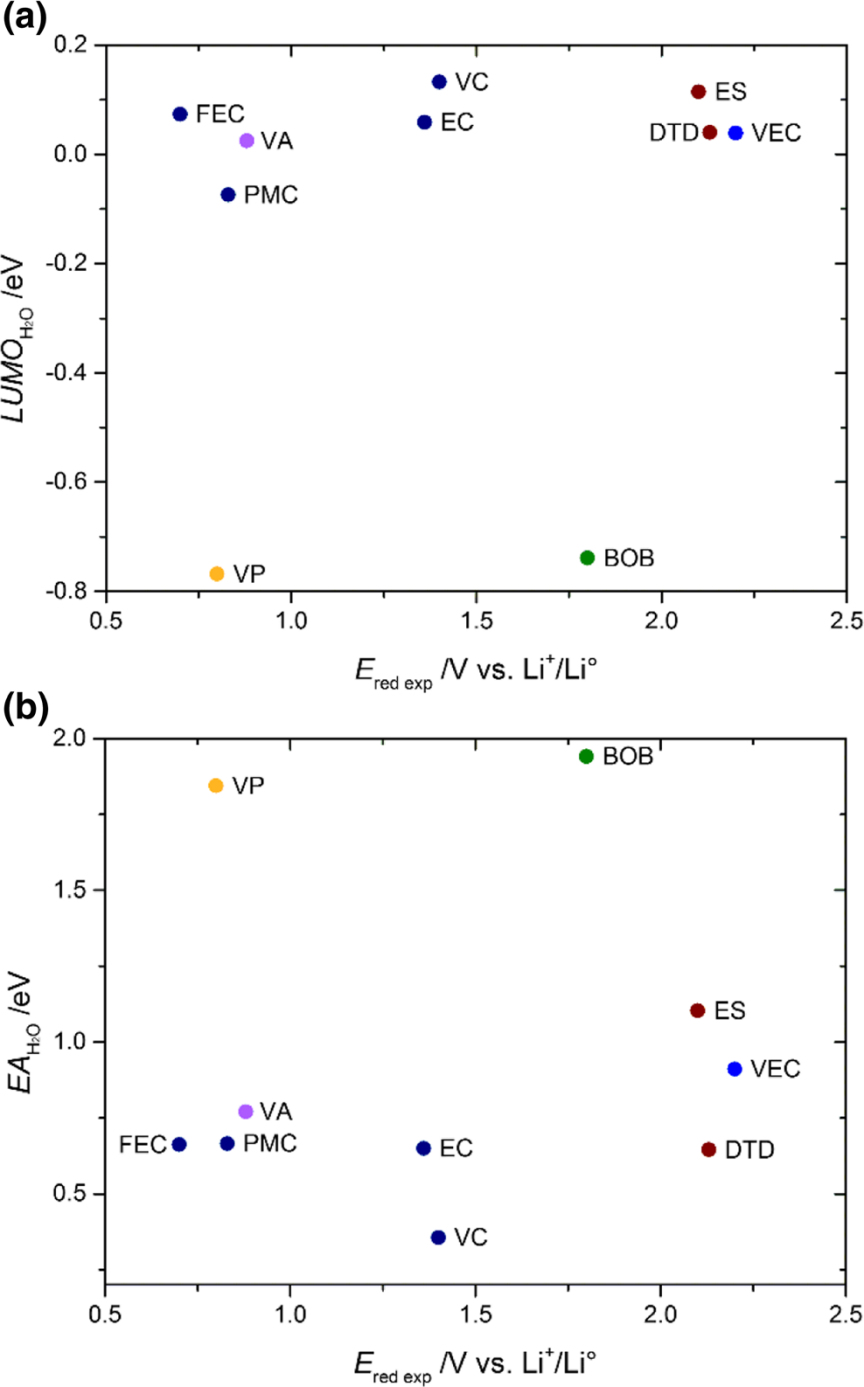
Relationship between the experimental reduction potential and **a** the LUMO energy or **b** the EA (both obtained using C-PCM M06-2X/6-311++G(d,p))

To facilitate the rapid screening of SEI-forming additives, it is advisable to find simple parameters that can easily be calculated for a large number of molecules. The most important properties should be the LUMO energy and the EA; however, based on our analysis (Fig. 5, Table 4), there were no significant trends in these properties. Therefore, descriptors are perhaps more suited to assessing the impact of small changes in the molecular structure, as already reported [73]. Comparisons of completely different molecules appear to be difficult when using a theoretical method that has been simplified by ignoring geometry relaxation, cation interactions, and so on.

**Table 4:** Dipole moments, HOMO and LUMO energies, ionization potentials, electron affinities, and binding energies to lithium cations as obtained using C-PCM M06-2X/6-311++G(d,p)

Additive	*μ* (D)	HOMO (eV)	LUMO (eV)	IP (eV)	EA (eV)	*η* (eV)	BE (kJ mol^−1^)
EC	7.33	−10.62	0.06	9.15	0.43	4.36	16.0
FEC	6.59	−11.02	0.07	9.55	0.66	4.44	11.4
VC	6.27	−8.74	0.13	7.62	0.36	3.63	14.1
VEC	7.63	−9.42	0.04	8.09	0.91	3.59	16.7
PMC	0.49	−9.58	−0.07	8.39	0.67	3.86	10.2
VA	2.31	−8.74	0.03	7.42	0.77	3.33	13.0
VP	2.82	−8.07	−0.77	6.96	1.84	2.56	19.1
ES	5.19	−9.92	0.11	8.57	1.10	3.73	15.3
DTD	7.98	−10.77	0.04	9.55	0.65	4.45	7.4
BOB	0.00	−9.71	−0.74	8.80	1.94	3.43	16.1

Useful information can be obtained by analyzing the chemical hardness, which—besides the HSAB concept [74]—is a viable measure of the tendency of a molecule to donate/accept an electron to/from an electrode. As a soft molecule can easily accept an electron and its reduced form is quite stable, the reduction of hard molecules immediately causes significant changes in geometry (e.g., bond cleavage). This is an important consideration when attempting to predict *E*_red_, as it requires the correct definition of the electrode reaction. As noted in Tables 2 and 3, for seven of the studied compounds, the cleavage of a chemical bond must occur to theoretically obtain reduction potentials similar to the experimentally derived values. It is very difficult to determine/guess if bond breaking should be considered to be part of the process that occurs at the electrode process for a completely new compound, but we have found that the chemical hardness is a very good descriptor. Additives softer than ca. 3.5 eV have stable reduced states and their path to SEI formation is determined by any electrode process; the electrode has no influence on the observed reduction potential. In contrast, the reduction of compounds harder than ca. 3.5 eV induces significant structural changes during the initial electrode-controlled process.

## Conclusions

Based on the analysis of ten SEI-forming compounds, we were able to find the most efficient procedure to predict their reduction behavior. The application of an implicit solvent was found to be necessary for any accurate prediction, but solvent permeability was not a crucial influence when it was >20. In addition, the lithium cation was observed to have a crucial influence on the thermodynamic cycle. Comparison of different methods and basis sets showed that functionals from the Minnesota family, especially M06-2X, were the best tools to describe the reduction potential. The popular Pople basis set 6-311++G(d,p) seems to be suitable, even though slightly better results can be obtained from the larger—and hence computationally more expensive—Dunning basis set aug-cc-pVTZ. Analysis of popular descriptors revealed that it is impossible to assess the reduction potential based on simple parameters such as the LUMO energy in a wide range of chemical compounds; such screening is only useful when considering compounds that show only small differences in structure. The chemical hardness was, however, found to be an useful property for predicting changes during the electrode process, even for very different chemistries.

## Electronic supplementary material

Below is the link to the electronic supplementary material.

ESM 1 (PDF 336 kb)

ESM 2 (RAR 73 kb)

## References

[1] Nishi Y (2001). J Power Sources.

[2] Armand M, Tarascon JM (2008). Nature.

[3] Scrosati B, Hassoun J, Sun YK (2011). Energy Environ Sci.

[4] Scrosati B, Garche J (2010). J Power Sources.

[5] Nitta N, Wu F, Lee JT, Yushin G (2014). Mater Today.

[6] Aurbach D, Ein-Eli Y (1995). J Electrochem Soc.

[7] Terada Y, Yasaka K, Nishikawa F, Konishi T, Yoshio M, Nakai I (2001). J Solid State Chem.

[8] Haregewoin AM, Wotangoa AS, Hwang BJ (2016). Energy Environ Sci.

[9] Aurbach D, Gamolsky K, Markovsky B, Gofer Y, Schmidt M, Heider U (2002). Electrochim Acta.

[10] Hu Y, Kong W, Li H, Huang X, Chen L (2004). Electrochem Commun.

[11] Ota H, Sakata Y, Inoue A, Yamahuchi S (2004). J Electrochem Soc.

[12] Sasaki T, Abe T, Iriyama Y, Inaba M, Ogumi Z (2005). J Electrochem Soc.

[13] Contestabile M, Morselli M, Paraventi R, Neat RJ (2003). J Power Sources.

[14] Li J, Yao W, Meng YS, Yang Y (2008). J Phys Chem C.

[15] Abe K, Hattori T, Kawabe K, Ushigoe Y, Uoshitake H (2007). J Electrochem Soc.

[16] Abe K, Miyoshi K, Hattori T, Ushigoe Y, Yoshitake H (2008). J Power Sources.

[17] Choi NS, Yew KH, Lee KY, Sung M, Kim H, Kim SS (2006). J Power Sources.

[18] Shu ZX, McMillan R, Murray JJ, Davidson I (1996). J Electrochem Soc.

[19] Janssen P, Schmitz R, Muller R, Isken P, Lex-Balducci A, Schreiner C, Winter M, Cekvic-Laskovic I, Schmitz R (2004). J Power Sources.

[20] Sano A, Maruyama S (2009). J Power Sources.

[21] Leung K (2013). Chem Phys Lett.

[22] Endo E, Ata M, Tanaka K, Sekai K (1998). J Electrochem Soc.

[23] Li T, Balbuena PB (2000). Chem Phys Lett.

[24] Wang Y, Balbuena PB (2002). J Phys Chem B.

[25] Wang Y, Nakamura S, Ue M, Balbuena PB (2001). J Am Chem Soc.

[26] Han YK, Lee SU (2004). Theor Chem Accounts.

[27] Wang Y, Nakamura S, Tasaki K, Balbuena PB (2002). J Am Chem Soc.

[28] Vollmer JM, Curtiss LA, Vissers DR, Amine K (2004). J Electrochem Soc.

[29] Leggesse EG, Jiang JC (2012). J Phys Chem A.

[30] Han YK, Lee SU, Ok JH, Cho JJ, Kim HJ (2002). Chem Phys Lett.

[31] Leggesse EG, Jiang JC (2012). RSC Adv.

[32] Husch T, Korth M (2015). Phys Chem Chem Phys.

[33] Cheng L, Assary RS, Qu X, Jain A, Ong SP, Rajput NN, Person K, Curtiss LA (2015). J Phys Chem Lett.

[34] Korth M (2015). Chem Model.

[35] Halls MD, Tasaki K (2010). J Power Sources.

[36] Park MH, Lee YS, Lee H, Han YK (2011). J Power Sources.

[37] Johansson P, Scheers J (2014) Prediction of electrolyte and additive electrochemical stabilities. In: Jow TR, Xu K, Borodin O, Ue M (ed) Electrolytes for lithium and lithium-ion batteries. Springer, New York

[38] Zhao Y, Schultz NE, Truhlar DG (2005). J Chem Phys.

[39] Zhao Y, Truhlar DG (2008). Theor Chem Accounts.

[40] Peverati R, Truhlar DG (2011). J Phys Chem Lett.

[41] Peverati R, Truhlar DG (2012). Phys Chem Chem Phys.

[42] Silva PJ, Ramos MJ (2011). Comput Theor Chem.

[43] Capobianco A, Vekardo A, Peluso A (2015). Comput Theor Chem.

[44] Jónsson E, Johansson P (2015). Phys Chem Chem Phys.

[45] Isegawa M, Neese F, Pantazis DA (2016). J Chem Theory Comput.

[46] Lespes N, Filhol JS (2015). J Chem Theory Comput.

[47] Aurbach D, Gofer Y, Ben-Zion M, Aped P (1992). J Electroanal Chem.

[48] Haregewoin AM, Leggesse EG, Jiang JC, Wang FM, Hwang BJ, Lin SD (2014). Electrochim Acta.

[49] Komaba S, Itabashi T, Ohtsuka T, Groult H, Kumagai N, Kaplan B, Yashiro H (2005). J Electrochem Soc.

[50] Frisch MJ, Trucks GW, Schlegel HB, Scuseria GE, Robb MA, Cheeseman JR, Scalmani G, Barone V, Mennucci B, Petersson GA, Nakatsuji H, Caricato M, Li X, Hratchian HP, Izmaylov AF, Bloino J, Zheng G, Sonnenberg JL, Hada M, Ehara M, Toyota K, Fukuda R, Hasegawa J, Ishida M, Nakajima T, Honda Y, Kitao O, Nakai H, Vreven T, Montgomery JA Jr, Peralta JE, Ogliaro F, Bearpark M, Heyd JJ, Brothers E, Kudin KN, Staroverov VN, Kobayashi R, Normand J, Raghavachari K, Rendell A, Burant JC, Iyengar SS, Tomasi J, Cossi M, Rega N, Millam JM, Klene M, Knox JE, Cross JB, Bakken V, Adamo C, Jaramillo J, Gomperts R, Stratmann RE, Yazyev O, Austin AJ, Cammi R, Pomelli C, Ochterski JW, Martin RL, Morokuma K, Zakrzewski VG, Voth GA, Salvador P, Dannenberg JJ, Dapprich S, Daniels AD, Farkas Ö, Foresman JB, Ortiz JV, Cioslowski J, Fox DJ (2009) Gaussian 09, revision E.01. Gaussian, Inc., Wallingford

[51] Schwabe T, Grimme S (2006). Phys Chem Chem Phys.

[52] Tao JM, Perdew JP, Staroverov VN, Scuseria GE (2003). Phys Rev Lett.

[53] Grimme S (2006). J Chem Phys.

[54] Becke AD (1993). J Chem Phys.

[55] Van Voorhis T, Scuseria GE (1998) J Chem Phys 109:400–410

[56] Adamo C, Barone V (1999). J Chem Phys.

[57] Zhao Y, Truhlar DG (2006). J Chem Phys.

[58] Krishnan R, Binkley JS, Seeger R, Pople JA (1980). J Chem Phys.

[59] Clark T, Chandrasekhar J, Spitznagel GW, Schleyer PVR (1983) J Comput Chem 4:294–301

[60] Rappoport D, Furche F (2010). J Chem Phys.

[61] Buczek A, Kupka T, Broda MA, Żyła A (2016). J Mol Model.

[62] Kendall RA, Dunning TH, Harrison RJ (1992). J Chem Phys.

[63] Barone V, Cossi M, Tomasi J (1998). J Comput Chem.

[64] Wang Y, Balbuena PB (2005). Int J Quantum Chem.

[65] Trasatti S (1986). Pure Appl Chem.

[66] Cramer CJ, Truhlar DG (1999). Chem Rev.

[67] Padmanabhan J, Parthasarathi R, Sarkar U, Subramanian V, Chattaraj PK (2004). Chem Phys Lett.

[68] Han YK, Lee K, Jung SC, Huk YS (2014). Comput Theor Chem.

[69] Zhang X, Kostecki R, Richardson TJ, Pugh JK, Ross PN (2001). J Electrochem Soc.

[70] Profatilova IA, Kim SS, Choi NS (2009). Electrochim Acta.

[71] Wrodnigg GH, Basenhard JO, Winter M (1999). J Electrochem Soc.

[72] Panitz JC, Wietelmann U, Wachtler M, Strobele S, Wohlfahrt-Mehrens M (2006). J Power Sources.

[73] Han YH, Moon Y, Lee K, Huh YS (2014). Curr Appl Phys.

[74] Pearson RG (1968). J Chem Educ.

