# Supplementary material for: SEI-forming electrolyte additives for lithium-ion batteries: development and benchmarking of computational approaches
Source: J Mol Model. 2016 Dec 13;23(1):6. doi: 10.1007/s00894-016-3180-0 (PMC5155019; doi:10.1007/s00894-016-3180-0)

ELECTRONIC SUPPLEMENTARY INFORMATION

SEI-forming Electrolyte Additives for Lithium-ion Batteries: Development and Benchmarking of Computational Approaches

Piotr Jankowski, Władysław Wieczorek, Patrik Johansson

Electronic energies and free energies of ground state geometries (start), reduced (red), vertically oxidized (vrtO) and vertically reduced (vrtR) – with and without a Li cation present [Hartree]

- BOB

E Li_red											
	HF	B2PLYP	B3LYP	M11	M06-2X	M06L	MN12L	mPW2PLY	PBE0	TPSSH	VSXC
H2O	-783.184	-785.922	-787.292	-787.020	-787.030	-787.205	-786.701	-785.985	-786.436	-787.290	-787.539
VACUUM	-783.059	-785.807	-787.179	-786.901	-786.912	-787.085	-786.581	-785.871	-786.323	-787.177	-787.421

G Li_red											
	HF	B2PLYP	B3LYP	M11	M06-2X	M06L	MN12L	mPW2PLY	PBE0	TPSSH	VSXC
H2O	-783.158	-786.630	-787.273	-786.997	-787.006	-787.183	-786.679	-786.631	-786.415	-787.272	-787.519
VACUUM	-783.031	-786.513	-787.155	-786.877	-786.887	-787.061	-786.556	-786.515	-786.298	-787.155	-787.400

E red											
	HF	B2PLYP	B3LYP	M11	M06-2X	M06L	MN12L	mPW2PLY	PBE0	TPSSH	VSXC
H2O	-775.735	-778.441	-779.795	-779.526	-779.531	-779.692	-779.176	-778.504	-778.962	-779.793	-780.019
VACUUM	-775.456	-778.165	-779.523	-779.252	-779.256	-779.422	-778.901	-778.228	-778.688	-779.521	-779.751

G red											
	HF	B2PLYP	B3LYP	M11	M06-2X	M06L	MN12L	mPW2PLY	PBE0	TPSSH	VSXC
H2O	-775.708	-779.150	-779.775	-779.505	-779.509	-779.672	-779.154	-779.151	-778.941	-779.774	-780.001
VACUUM	-775.429	-778.877	-779.503	-779.231	-779.234	-779.402	-778.879	-778.878	-778.667	-779.502	-779.734

E Li_start											
	HF	B2PLYP	B3LYP	M11	M06-2X	M06L	MN12L	mPW2PLY	PBE0	TPSSH	VSXC
H2O	-783.106	-785.823	-787.178	-786.900	-786.915	-787.099	-786.598	-785.884	-786.326	-787.180	-787.427
VACUUM	-783.026	-785.746	-787.105	-786.826	-786.841	-787.023	-786.521	-785.808	-786.252	-787.107	-787.354

G Li_start											
	HF	B2PLYP	B3LYP	M11	M06-2X	M06L	MN12L	mPW2PLY	PBE0	TPSSH	VSXC
H2O	-783.076	-786.523	-787.154	-786.875	-786.889	-787.076	-786.572	-786.523	-786.301	-787.157	-787.405
VACUUM	-782.993	-786.447	-787.079	-786.799	-786.813	-786.999	-786.494	-786.448	-786.225	-787.082	-787.330

E start											
	HF	B2PLYP	B3LYP	M11	M06-2X	M06L	MN12L	mPW2PLY	PBE0	TPSSH	VSXC
H2O	-775.670	-778.354	-779.694	-779.424	-779.434	-779.601	-779.090	-778.416	-778.864	-779.695	-779.923
VACUUM	-775.594	-778.280	-779.621	-779.351	-779.361	-779.529	-779.017	-778.342	-778.792	-779.624	-779.851

G start											
	HF	B2PLYP	B3LYP	M11	M06-2X	M06L	MN12L	mPW2PLY	PBE0	TPSSH	VSXC
H2O	-775.640	-779.056	-779.670	-779.399	-779.408	-779.578	-779.064	-779.056	-778.839	-779.672	-779.901
VACUUM	-775.564	-778.983	-779.598	-779.326	-779.335	-779.506	-778.991	-778.983	-778.767	-779.601	-779.829

E vrtO											
	HF	B2PLYP	B3LYP	M11	M06-2X	M06L	MN12L	mPW2PLY	PBE0	TPSSH	VSXC
H2O					-779.110						
VACUUM					-779.099						

E vrtR											
	HF	B2PLYP	B3LYP	M11	M06-2X	M06L	MN12L	mPW2PLY	PBE0	TPSSH	VSXC
H2O					-779.505						
VACUUM					-779.228						

- DTD

E Li_red											
	HF	B2PLYP	B3LYP	M11	M06-2X	M06L	MN12L	mPW2PLY	PBE0	TPSSH	VSXC
THF					-785.184						
ACT					-785.190						
AN					-785.191						
H2O	-782.627	-784.479	-785.387	-785.203	-785.192	-785.326	-784.990	-784.527	-784.793	-785.390	-785.655
VACUUM	-782.572	-784.426	-785.331	-785.148	-785.138	-785.265	-784.932	-784.477	-784.737	-785.333	-785.597

G Li_red											
	HF	B2PLYP	B3LYP	M11	M06-2X	M06L	MN12L	mPW2PLY	PBE0	TPSSH	VSXC
					-785.147						
ACT					-785.153						
AN					-785.156						
H2O	-775.140	-784.853	-785.352	-785.167	-785.155	-785.290	-784.953	-784.865	-784.756	-785.355	-785.623
VACUUM	-775.042	-784.797	-785.296	-785.112	-785.100	-785.228	-784.894	-784.812	-784.701	-785.298	-785.562

E red											
	HF	B2PLYP	B3LYP	M11	M06-2X	M06L	MN12L	mPW2PLY	PBE0	TPSSH	VSXC
THF					-777.688						
ACT					-777.698						
AN					-777.700						
H2O	-775.181	-777.000	-777.897	-777.718	-777.702	-777.826	-777.478	-777.049	-777.326	-777.901	-778.151
VACUUM	-775.083	-776.895	-777.808	-777.568	-777.605	-777.736	-777.334	-776.942	-777.232	-777.810	

G red											
	HF	B2PLYP	B3LYP	M11	M06-2X	M06L	MN12L	mPW2PLY	PBE0	TPSSH	VSXC
THF					-777.650						
ACT					-777.663						
AN					-777.664						
H2O	-775.031	-777.380	-777.864	-777.683	-777.665	-777.789	-777.440	-777.392	-777.289	-777.865	-778.119
VACUUM	-774.974	-777.297	-777.778	-777.523	-777.571	-777.700	-777.290	-777.305	-777.199	-777.774	-777.944

E Li_start											
	HF	B2PLYP	B3LYP	M11	M06-2X	M06L	MN12L	mPW2PLY	PBE0	TPSSH	VSXC
THF					-785.045						
ACT					-785.058						
AN					-785.062						
H2O	-782.499	-784.349	-785.244	-785.070	-785.064	-785.196	-784.874	-784.397	-784.666	-785.256	-785.504
VACUUM	-782.357	-784.208	-785.103	-784.928	-784.922	-785.052	-784.732	-784.256	-784.524	-785.114	-785.361

G Li_start											
	HF	B2PLYP	B3LYP	M11	M06-2X	M06L	MN12L	mPW2PLY	PBE0	TPSSH	VSXC
THF					-785.000						
ACT					-785.013						
AN					-785.017						
H2O	-782.449	-784.716	-785.202	-785.025	-785.019	-785.153	-784.829	-784.728	-784.621	-785.213	-785.462
VACUUM	-782.306	-784.574	-785.060	-784.884	-784.877	-785.008	-784.687	-784.586	-784.479	-785.071	-785.319

E start											
	HF	B2PLYP	B3LYP	M11	M06-2X	M06L	MN12L	mPW2PLY	PBE0	TPSSH	VSXC
THF					-777.584						
ACT					-777.586						
AN					-777.586						
H2O	-775.065	-776.884	-777.763	-777.597	-777.586	-777.702	-777.370	-776.932	-777.208	-777.775	-778.007
VACUUM	-775.046	-776.867	-777.749	-777.581	-777.571	-777.689	-777.355	-776.915	-777.194	-777.762	-777.994

G start											
	HF	B2PLYP	B3LYP	M11	M06-2X	M06L	MN12L	mPW2PLY	PBE0	TPSSH	VSXC
THF					-777.538						
ACT					-777.540						
AN					-777.540						
H2O	-775.015	-777.251	-777.719	-777.553	-777.540	-777.658	-777.325	-777.264	-777.163	-777.732	-777.964
VACUUM	-774.996	-777.237	-777.705	-777.538	-777.525	-777.646	-777.308	-777.249	-777.149	-777.718	-777.951

E vrtO											
	HF	B2PLYP	B3LYP	M11	M06-2X	M06L	MN12L	mPW2PLY	PBE0	TPSSH	VSXC
THF					-777.223						
ACT					-777.232						
AN					-777.232						
H2O					-777.235						
VACUUM					-777.137						

E vrtR											
	HF	B2PLYP	B3LYP	M11	M06-2X	M06L	MN12L	mPW2PLY	PBE0	TPSSH	VSXC
THF					-777.597						
ACT					-777.606						
AN					-777.608						
H2O					-777.610						
VACUUM					-777.554						

- EC

E Li_red	HF	B2PLYP	B3LYP	M11	M06-2X	M06L	MN12L	mPW2PLY	PBE0	TPSSH	VSXC
THF					-349.962						
ACT					-349.967						
AN					-349.968						
H2O	-348.174	-349.458	-350.112	-349.958	-349.969	-350.068	-349.804	-349.487	-349.706	-350.112	-350.233
VACUUM	-348.122	-349.408	-350.062	-349.907	-349.919	-350.017	-349.751	-349.437	-349.656	-350.062	-350.182

G Li_red	HF	B2PLYP	B3LYP	M11	M06-2X	M06L	MN12L	mPW2PLY	PBE0	TPSSH	VSXC
THF					-349.923						
ACT					-349.928						
AN					-349.930						
H2O	-348.132	-349.755	-350.076	-349.922	-349.931	-350.032	-349.766	-349.756	-349.670	-350.076	-350.195
VACUUM	-348.078	-349.702	-350.024	-349.869	-349.879	-349.977	-349.712	-349.703	-349.617	-350.024	-350.143

E red	HF	B2PLYP	B3LYP	M11	M06-2X	M06L	MN12L	mPW2PLY	PBE0	TPSSH	VSXC
THF					-342.461						
ACT					-342.470						
AN					-342.473						
H2O	-340.665	-341.976	-342.615	-342.468	-342.474	-342.559	-342.283	-342.006	-342.231	-342.614	-342.717
VACUUM	-340.601	-341.874	-342.516	-342.366	-342.373	-342.460	-342.182	-341.902	-342.131	-342.515	-342.617
					-342.461						

G red	HF	B2PLYP	B3LYP	M11	M06-2X	M06L	MN12L	mPW2PLY	PBE0	TPSSH	VSXC
THF					-342.424						
ACT					-342.433						
AN					-342.435						
H2O	-340.618	-342.275	-342.580	-342.432	-342.437	-342.523	-342.246	-342.275	-342.195	-342.580	-342.681
VACUUM	-340.549	-342.176	-342.482	-342.329	-342.335	-342.424	-342.145	-342.174	-342.097	-342.480	-342.582

E Li_start	HF	B2PLYP	B3LYP	M11	M06-2X	M06L	MN12L	mPW2PLY	PBE0	TPSSH	VSXC
THF					-349.848						
ACT					-349.860						
AN					-349.863						
H2O	-348.081	-349.356	-350.003	-349.850	-349.865	-349.966	-349.711	-349.385	-349.604	-350.009	-350.114
VACUUM	-347.948	-349.224	-349.871	-349.718	-349.733	-349.833	-349.579	-349.253	-349.472	-349.876	-349.980

G Li_start	HF	B2PLYP	B3LYP	M11	M06-2X	M06L	MN12L	mPW2PLY	PBE0	TPSSH	VSXC
THF					-349.800						
ACT					-349.812						
AN					-349.815						
H2O	-348.028	-349.641	-349.956	-349.804	-349.817	-349.920	-349.663	-349.641	-349.557	-349.963	-350.068
VACUUM	-347.896	-349.508	-349.824	-349.672	-349.685	-349.786	-349.531	-349.509	-349.425	-349.830	-349.935

E start	HF	B2PLYP	B3LYP	M11	M06-2X	M06L	MN12L	mPW2PLY	PBE0	TPSSH	VSXC
THF					-342.382						
ACT					-349.860						
AN					-342.384						
H2O	-340.644	-341.887	-342.517	-342.374	-342.382	-342.469	-342.203	-341.916	-342.143	-342.524	-342.613
VACUUM	-340.627	-341.872	-342.503	-342.359	-342.370	-342.457	-342.189	-341.901	-342.129	-342.511	-342.600

G start	HF	B2PLYP	B3LYP	M11	M06-2X	M06L	MN12L	mPW2PLY	PBE0	TPSSH	VSXC
THF					-342.334						
ACT					-342.336						
AN					-342.336						
H2O	-340.591	-342.173	-342.471	-342.328	-342.336	-342.423	-342.155	-342.174	-342.095	-342.478	-342.568
VACUUM	-340.575	-342.160	-342.457	-342.313	-342.322	-342.410	-342.141	-342.160	-342.082	-342.465	-342.554

E vrtO	HF	B2PLYP	B3LYP	M11	M06-2X	M06L	MN12L	mPW2PLY	PBE0	TPSSH	VSXC
THF					-342.026						
ACT					-342.036						
AN					-342.038						
H2O					-342.040						
VACUUM					-341.950						

E vrtR	HF	B2PLYP	B3LYP	M11	M06-2X	M06L	MN12L	mPW2PLY	PBE0	TPSSH	VSXC
THF					-342.398						
ACT					-342.405						
AN					-342.407						
H2O					-342.408						
VACUUM					-342.331						

- ES

E Li_red											
	HF	B2PLYP	B3LYP	M11	M06-2X	M06L	MN12L	mPW2PLY	PBE0	TPSSH	VSXC
H2O	-783.184	-709.366	-710.159	-709.998	-709.989	-710.098	-709.817	-709.409	-709.638	-710.159	-710.407
VACUUM	-783.059	-709.332	-710.126	-709.965	-709.957	-710.064	-709.784	-709.375	-709.605	-710.127	-710.372

G Li_red											
	HF	B2PLYP	B3LYP	M11	M06-2X	M06L	MN12L	mPW2PLY	PBE0	TPSSH	VSXC
H2O	-783.158	-709.683	-710.126	-709.964	-709.955	-710.065	-709.782	-709.695	-709.603	-710.125	-710.372
VACUUM	-783.031	-709.647	-710.090	-709.928	-709.919	-710.027	-709.746	-709.660	-709.568	-710.090	-710.336

E red											
	HF	B2PLYP	B3LYP	M11	M06-2X	M06L	MN12L	mPW2PLY	PBE0	TPSSH	VSXC
H2O	-783.184	-701.873	-702.655	-702.499	-702.486	-702.584	-702.292	-701.916	-702.158	-702.656	-702.881
VACUUM	-783.059	-701.778	-702.562	-702.362	-702.391	-702.492	-702.200	-701.820	-702.064	-702.564	-702.788

G red											
	HF	B2PLYP	B3LYP	M11	M06-2X	M06L	MN12L	mPW2PLY	PBE0	TPSSH	VSXC
H2O	-775.708	-702.195	-702.621	-702.466	-702.451	-702.551	-702.257	-702.207	-702.123	-702.623	-702.849
VACUUM	-775.429	-702.104	-702.530	-702.323	-702.357	-702.460	-702.166	-702.114	-702.031	-702.532	-702.757

E Li_start											
	HF	B2PLYP	B3LYP	M11	M06-2X	M06L	MN12L	mPW2PLY	PBE0	TPSSH	VSXC
H2O	-783.184	-709.248	-710.026	-709.871	-709.868	-709.975	-709.703	-709.289	-709.516	-710.034	-710.263
VACUUM	-783.059	-709.116	-709.893	-709.738	-709.735	-709.839	-709.569	-709.158	-709.383	-709.900	-710.127

G Li_start											
	HF	B2PLYP	B3LYP	M11	M06-2X	M06L	MN12L	mPW2PLY	PBE0	TPSSH	VSXC
H2O	-783.076	-709.554	-709.986	-709.830	-709.826	-709.936	-709.662	-709.565	-709.475	-709.995	-710.224
VACUUM	-782.993	-709.420	-709.854	-709.698	-709.693	-709.800	-709.528	-709.432	-709.342	-709.861	-710.089

E start											
	HF	B2PLYP	B3LYP	M11	M06-2X	M06L	MN12L	mPW2PLY	PBE0	TPSSH	VSXC
H2O	-775.670	-701.779	-702.543	-702.395	-702.387	-702.479	-702.196	-701.821	-702.056	-702.551	-702.764
VACUUM	-775.594	-701.767	-702.532	-702.384	-702.376	-702.470	-702.186	-701.809	-702.046	-702.541	-702.755

G start											
	HF	B2PLYP	B3LYP	M11	M06-2X	M06L	MN12L	mPW2PLY	PBE0	TPSSH	VSXC
H2O	-775.640	-702.088	-702.504	-702.355	-702.346	-702.440	-702.155	-702.100	-702.016	-702.512	-702.726
VACUUM	-775.564	-702.077	-702.493	-702.344	-702.335	-702.431	-702.145	-702.089	-702.006	-702.502	-702.716

E vrtO											
	HF	B2PLYP	B3LYP	M11	M06-2X	M06L	MN12L	mPW2PLY	PBE0	TPSSH	VSXC
H2O					-702.072						
VACUUM					-701.976						

E vrtR											
	HF	B2PLYP	B3LYP	M11	M06-2X	M06L	MN12L	mPW2PLY	PBE0	TPSSH	VSXC
H2O					-702.427						
VACUUM					-702.350						

- FEC

E Li_red											
	HF	B2PLYP	B3LYP	M11	M06-2X	M06L	MN12L	mPW2PLY	PBE0	TPSSH	VSXC
H2O	-447.034	-448.582	-449.362	-449.198	-449.188	-449.305	-449.005	-448.617	-448.865	-449.356	-449.515
VACUUM	-446.900	-448.447	-449.227	-449.064	-449.053	-449.170	-448.871	-448.482	-448.730	-449.221	-449.380

G Li_red											
	HF	B2PLYP	B3LYP	M11	M06-2X	M06L	MN12L	mPW2PLY	PBE0	TPSSH	VSXC
H2O	-447.000	-448.953	-449.333	-449.169	-449.157	-449.276	-448.975	-448.953	-448.836	-449.328	-449.486
VACUUM	-446.866	-448.818	-449.199	-449.034	-449.022	-449.141	-448.840	-448.818	-448.702	-449.193	-449.351

E red											
	HF	B2PLYP	B3LYP	M11	M06-2X	M06L	MN12L	mPW2PLY	PBE0	TPSSH	VSXC
H2O	-439.596	-441.112	-441.875	-441.720	-441.705	-441.805	-441.495	-441.147	-441.401	-441.870	-442.012
VACUUM	-439.521	-441.037	-441.800	-441.645	-441.629	-441.730	-441.420	-441.072	-441.326	-441.795	-441.937

G red											
	HF	B2PLYP	B3LYP	M11	M06-2X	M06L	MN12L	mPW2PLY	PBE0	TPSSH	VSXC
H2O	-439.562	-441.482	-441.848	-441.690	-441.673	-441.776	-441.464	-441.484	-441.371	-441.842	-441.984
VACUUM	-439.487	-441.407	-441.772	-441.615	-441.598	-441.701	-441.389	-441.408	-441.296	-441.766	-441.909

E Li_start											
	HF	B2PLYP	B3LYP	M11	M06-2X	M06L	MN12L	mPW2PLY	PBE0	TPSSH	VSXC
H2O	-446.969	-448.506	-449.276	-449.111	-449.113	-449.227	-448.934	-448.541	-448.789	-449.278	-449.412
VACUUM	-446.831	-448.368	-449.137	-448.972	-448.975	-449.089	-448.795	-448.403	-448.650	-449.140	-449.274

G Li_start											
	HF	B2PLYP	B3LYP	M11	M06-2X	M06L	MN12L	mPW2PLY	PBE0	TPSSH	VSXC
H2O	-446.926	-448.865	-449.239	-449.073	-449.074	-449.190	-448.895	-448.866	-448.751	-449.242	-449.376
VACUUM	-446.788	-448.727	-449.101	-448.935	-448.936	-449.051	-448.757	-448.728	-448.613	-449.103	-449.238

E start											
	HF	B2PLYP	B3LYP	M11	M06-2X	M06L	MN12L	mPW2PLY	PBE0	TPSSH	VSXC
H2O	-439.534	-441.039	-441.792	-441.636	-441.633	-441.731	-441.427	-441.074	-441.329	-441.795	-441.913
VACUUM	-439.521	-441.026	-441.779	-441.623	-441.620	-441.718	-441.414	-441.061	-441.316	-441.782	-441.900

G start											
	HF	B2PLYP	B3LYP	M11	M06-2X	M06L	MN12L	mPW2PLY	PBE0	TPSSH	VSXC
H2O	-439.491	-441.399	-441.755	-441.599	-441.595	-441.694	-441.389	-441.400	-441.291	-441.758	-441.877
VACUUM	-439.477	-441.385	-441.742	-441.585	-441.581	-441.681	-441.375	-441.386	-441.277	-441.745	-441.864

E vrtO											
	HF	B2PLYP	B3LYP	M11	M06-2X	M06L	MN12L	mPW2PLY	PBE0	TPSSH	VSXC
H2O					-441.283						
VACUUM					-441.182						

E vrtR											
	HF	B2PLYP	B3LYP	M11	M06-2X	M06L	MN12L	mPW2PLY	PBE0	TPSSH	VSXC
H2O					-441.658						
VACUUM					-441.599						

- PMC

E Li_red											
	HF	B2PLYP	B3LYP	M11	M06-2X	M06L	MN12L	mPW2PLY	PBE0	TPSSH	VSXC
H2O	-425.023	-426.643	-427.474	-427.259	-427.289	-427.412	-427.076	-426.679	-426.958	-427.472	-427.628
VACUUM	-424.996	-426.622	-427.454	-427.238	-427.268	-427.390	-427.054	-426.659	-426.938	-427.452	-427.607

G Li_red											
	HF	B2PLYP	B3LYP	M11	M06-2X	M06L	MN12L	mPW2PLY	PBE0	TPSSH	VSXC
H2O	-424.970	-427.017	-427.425	-427.201	-427.236	-427.356	-427.018	-427.018	-426.907	-427.422	-427.571
VACUUM	-424.943	-426.997	-427.405	-427.180	-427.214	-427.335	-426.996	-426.998	-426.886	-427.402	-427.551

E red											
	HF	B2PLYP	B3LYP	M11	M06-2X	M06L	MN12L	mPW2PLY	PBE0	TPSSH	VSXC
H2O	-417.505	-419.185	-420.002	-419.795	-419.817	-419.929	-419.584	-419.221	-419.510	-420.001	-420.152
VACUUM	-417.444	-419.131	-419.950	-419.741	-419.762	-419.874	-419.530	-419.168	-419.456	-419.948	-420.098

G red											
	HF	B2PLYP	B3LYP	M11	M06-2X	M06L	MN12L	mPW2PLY	PBE0	TPSSH	VSXC
H2O	-417.427	-419.522	-419.928	-419.714	-419.742	-419.850	-419.502	-419.536	-419.437	-419.924	-420.059
VACUUM	-417.366	-419.468	-419.876	-419.660	-419.687	-419.796	-419.447	-419.482	-419.383	-419.871	-420.005

E Li_start											
	HF	B2PLYP	B3LYP	M11	M06-2X	M06L	MN12L	mPW2PLY	PBE0	TPSSH	VSXC
H2O	-424.929	-426.543	-427.367	-427.161	-427.194	-427.315	-426.990	-426.580	-426.861	-427.371	-427.518
VACUUM	-424.788	-426.403	-427.228	-427.020	-427.053	-427.174	-426.849	-426.440	-426.721	-427.232	-427.377

G Li_start											
	HF	B2PLYP	B3LYP	M11	M06-2X	M06L	MN12L	mPW2PLY	PBE0	TPSSH	VSXC
H2O	-424.850	-426.899	-427.298	-427.090	-427.122	-427.246	-426.918	-426.899	-426.791	-427.303	-427.449
VACUUM	-424.709	-426.759	-427.159	-426.950	-426.982	-427.104	-426.777	-426.759	-426.651	-427.163	-427.309

E start											
	HF	B2PLYP	B3LYP	M11	M06-2X	M06L	MN12L	mPW2PLY	PBE0	TPSSH	VSXC
H2O	-417.494	-419.076	-419.883	-419.686	-419.714	-419.819	-419.483	-419.113	-419.401	-419.888	-420.018
VACUUM	-417.483	-419.066	-419.873	-419.677	-419.704	-419.811	-419.474	-419.103	-419.392	-419.879	-420.009

G start											
	HF	B2PLYP	B3LYP	M11	M06-2X	M06L	MN12L	mPW2PLY	PBE0	TPSSH	VSXC
H2O	-417.415	-419.433	-419.814	-419.616	-419.642	-419.752	-419.412	-419.433	-419.331	-419.819	-419.949
VACUUM	-417.404	-419.423	-419.804	-419.607	-419.632	-419.740	-419.402	-419.423	-419.321	-419.810	-419.940

E vrtO											
	HF	B2PLYP	B3LYP	M11	M06-2X	M06L	MN12L	mPW2PLY	PBE0	TPSSH	VSXC
H2O					-419.405						
VACUUM					-419.310						

E vrtR											
	HF	B2PLYP	B3LYP	M11	M06-2X	M06L	MN12L	mPW2PLY	PBE0	TPSSH	VSXC
H2O					-419.738						
VACUUM					-419.676						

- VA

E Li_red											
	HF	B2PLYP	B3LYP	M11	M06-2X	M06L	MN12L	mPW2PLY	PBE0	TPSSH	VSXC
H2O	-312.213	-313.493	-314.133	-313.965	-313.988	-314.085	-313.830	-313.522	-313.748	-314.135	-314.245
VACUUM	-312.216	-313.455	-314.077	-313.915	-313.938	-314.021	-313.777	-313.470	-313.692	-314.077	-314.190

G Li_red											
	HF	B2PLYP	B3LYP	M11	M06-2X	M06L	MN12L	mPW2PLY	PBE0	TPSSH	VSXC
H2O	-312.190	-313.757	-314.074	-313.906	-313.927	-314.026	-313.770	-313.757	-313.688	-314.076	-314.188
VACUUM	-312.149	-313.723	-314.018	-313.855	-313.876	-313.961	-313.715	-313.704	-313.632	-314.018	-314.132

E red											
	HF	B2PLYP	B3LYP	M11	M06-2X	M06L	MN12L	mPW2PLY	PBE0	TPSSH	VSXC
H2O	-304.785	-306.008	-306.634	-306.474	-306.491	-306.575	-306.309	-306.037	-306.272	-306.636	-306.733
VACUUM	-304.729	-305.925	-306.548	-306.382	-306.399	-306.489	-306.231	-305.953	-306.186	-306.549	-306.653

G red											
	HF	B2PLYP	B3LYP	M11	M06-2X	M06L	MN12L	mPW2PLY	PBE0	TPSSH	VSXC
H2O	-304.712	-306.275	-306.576	-306.415	-306.432	-306.517	-306.249	-306.276	-306.213	-306.578	-306.677
VACUUM	-304.660	-306.187	-306.490	-306.323	-306.338	-306.431	-306.170	-306.188	-306.126	-306.492	-306.595

E Li_start											
	HF	B2PLYP	B3LYP	M11	M06-2X	M06L	MN12L	mPW2PLY	PBE0	TPSSH	VSXC
H2O	-312.210	-313.430	-314.058	-313.890	-313.917	-314.020	-313.769	-313.458	-313.676	-314.061	-314.174
VACUUM	-312.075	-313.297	-313.924	-313.756	-313.783	-313.884	-313.634	-313.324	-313.541	-313.927	-314.039

G Li_start											
	HF	B2PLYP	B3LYP	M11	M06-2X	M06L	MN12L	mPW2PLY	PBE0	TPSSH	VSXC
H2O	-312.140	-313.685	-313.995	-313.826	-313.853	-313.956	-313.704	-313.685	-313.612	-313.999	-314.113
VACUUM	-312.006	-313.548	-313.861	-313.691	-313.718	-313.821	-313.570	-313.549	-313.478	-313.864	-313.975

E start											
	HF	B2PLYP	B3LYP	M11	M06-2X	M06L	MN12L	mPW2PLY	PBE0	TPSSH	VSXC
H2O	-304.774	-305.962	-306.573	-306.414	-306.437	-306.523	-306.261	-305.990	-306.215	-306.577	-306.673
VACUUM	-304.767	-305.956	-306.567	-306.408	-306.431	-306.518	-306.255	-305.983	-306.209	-306.571	-306.668

G start											
	HF	B2PLYP	B3LYP	M11	M06-2X	M06L	MN12L	mPW2PLY	PBE0	TPSSH	VSXC
H2O	-304.706	-306.219	-306.511	-306.353	-306.372	-306.460	-306.197	-306.219	-306.152	-306.515	-306.613
VACUUM	-304.697	-306.213	-306.505	-306.344	-306.366	-306.455	-306.191	-306.213	-306.146	-306.509	-306.607

E vrtO											
	HF	B2PLYP	B3LYP	M11	M06-2X	M06L	MN12L	mPW2PLY	PBE0	TPSSH	VSXC
H2O					-306.164						
VACUUM					-306.072						

E vrtR											
	HF	B2PLYP	B3LYP	M11	M06-2X	M06L	MN12L	mPW2PLY	PBE0	TPSSH	VSXC
H2O					-306.465						
VACUUM					-306.399						

- VC

E Li_red	HF	B2PLYP	B3LYP	M11	M06-2X	M06L	MN12L	mPW2PLY	PBE0	TPSSH	VSXC
H2O	-346.962	-348.219	-348.858	-348.710	-348.723	-348.821	-348.574	-348.246	-348.455	-348.858	-348.979
VACUUM	-346.909	-348.166	-348.805	-348.656	-348.670	-348.766	-348.520	-348.194	-348.402	-348.805	-348.925

G Li_red	HF	B2PLYP	B3LYP	M11	M06-2X	M06L	MN12L	mPW2PLY	PBE0	TPSSH	VSXC
H2O	-346.944	-348.531	-348.843	-348.694	-348.707	-348.805	-348.558	-348.531	-348.440	-348.843	-348.965
VACUUM	-346.889	-348.477	-348.789	-348.640	-348.653	-348.749	-348.502	-348.477	-348.385	-348.789	-348.910

E red	HF	B2PLYP	B3LYP	M11	M06-2X	M06L	MN12L	mPW2PLY	PBE0	TPSSH	VSXC
H2O	-339.516	-340.740	-341.365	-341.224	-341.231	-341.315	-341.057	-340.768	-340.984	-341.364	-341.468
VACUUM	-339.414	-340.641	-341.269	-341.125	-341.133	-341.221	-340.961	-340.669	-340.888	-341.269	-341.374

G red	HF	B2PLYP	B3LYP	M11	M06-2X	M06L	MN12L	mPW2PLY	PBE0	TPSSH	VSXC
H2O	-339.499	-341.056	-341.351	-341.210	-341.217	-341.302	-341.043	-341.055	-340.970	-341.351	-341.455
VACUUM	-339.397	-340.960	-341.257	-341.112	-341.119	-341.209	-340.947	-340.960	-340.874	-341.256	-341.364

E Li_start	HF	B2PLYP	B3LYP	M11	M06-2X	M06L	MN12L	mPW2PLY	PBE0	TPSSH	VSXC
H2O	-346.878	-348.132	-348.765	-348.620	-348.635	-348.734	-348.498	-348.159	-348.371	-348.772	-348.882
VACUUM	-346.744	-347.999	-348.631	-348.486	-348.501	-348.598	-348.364	-348.026	-348.237	-348.637	-348.747

G Li_start	HF	B2PLYP	B3LYP	M11	M06-2X	M06L	MN12L	mPW2PLY	PBE0	TPSSH	VSXC
H2O	-346.850	-348.437	-348.741	-348.595	-348.610	-348.710	-348.473	-348.435	-348.346	-348.748	-348.859
VACUUM	-346.717	-348.303	-348.608	-348.463	-348.477	-348.574	-348.339	-348.302	-348.213	-348.615	-348.725

E start	HF	B2PLYP	B3LYP	M11	M06-2X	M06L	MN12L	mPW2PLY	PBE0	TPSSH	VSXC
H2O	-339.443	-340.664	-341.281	-341.145	-341.155	-341.237	-340.991	-340.692	-340.911	-341.288	-341.383
VACUUM	-339.430	-340.653	-341.271	-341.134	-341.144	-341.228	-340.980	-340.680	-340.901	-341.278	-341.373

G start	HF	B2PLYP	B3LYP	M11	M06-2X	M06L	MN12L	mPW2PLY	PBE0	TPSSH	VSXC
H2O	-339.415	-340.971	-341.258	-341.122	-341.131	-341.214	-340.967	-340.970	-340.887	-341.265	-341.361
VACUUM	-339.402	-340.961	-341.248	-341.110	-341.121	-341.205	-340.956	-340.960	-340.877	-341.256	-341.351

E vrtO	HF	B2PLYP	B3LYP	M11	M06-2X	M06L	MN12L	mPW2PLY	PBE0	TPSSH	VSXC
H2O					-340.874						
VACUUM					-340.772						

E vrtR	HF	B2PLYP	B3LYP	M11	M06-2X	M06L	MN12L	mPW2PLY	PBE0	TPSSH	VSXC
H2O					-341.168						
VACUUM					-341.083						

- VEC

E Li_red											
	HF	B2PLYP	B3LYP	M11	M06-2X	M06L	MN12L	mPW2PLY	PBE0	TPSSH	VSXC
H2O	-425.102	-426.724	-427.556	-427.347	-427.374	-427.498	-427.165	-426.762	-427.052	-427.559	-427.714
VACUUM	-425.049	-426.673	-427.505	-427.295	-427.323	-427.446	-427.113	-426.711	-427.001	-427.508	-427.663

G Li_red											
	HF	B2PLYP	B3LYP	M11	M06-2X	M06L	MN12L	mPW2PLY	PBE0	TPSSH	VSXC
H2O	-425.027	-427.080	-427.489	-427.279	-427.304	-427.430	-427.096	-427.081	-426.983	-427.492	-427.646
VACUUM	-424.973	-427.027	-427.436	-427.225	-427.251	-427.376	-427.042	-427.029	-426.931	-427.439	-427.593

E red											
	HF	B2PLYP	B3LYP	M11	M06-2X	M06L	MN12L	mPW2PLY	PBE0	TPSSH	VSXC
H2O	-417.653	-419.243	-420.059	-419.857	-419.879	-419.989	-419.645	-419.281	-419.577	-420.061	-420.198
VACUUM	-417.547	-419.142	-419.959	-419.757	-419.780	-419.892	-419.547	-419.131	-419.479	-419.963	-420.101

G red											
	HF	B2PLYP	B3LYP	M11	M06-2X	M06L	MN12L	mPW2PLY	PBE0	TPSSH	VSXC
H2O	-417.579	-419.600	-419.992	-419.790	-419.811	-419.922	-419.577	-419.602	-419.510	-419.995	-420.131
VACUUM	-417.475	-419.503	-419.895	-419.691	-419.712	-419.826	-419.480	-419.450	-419.413	-419.897	-420.035

E Li_start											
	HF	B2PLYP	B3LYP	M11	M06-2X	M06L	MN12L	mPW2PLY	PBE0	TPSSH	VSXC
H2O	-424.981	-426.597	-427.421	-427.214	-427.247	-427.370	-427.046	-426.634	-426.924	-427.430	-427.567
VACUUM	-424.852	-426.468	-427.291	-427.084	-427.117	-427.239	-426.916	-426.506	-426.795	-427.301	-427.436

G Li_start											
	HF	B2PLYP	B3LYP	M11	M06-2X	M06L	MN12L	mPW2PLY	PBE0	TPSSH	VSXC
H2O	-424.897	-426.946	-427.345	-427.138	-427.170	-427.295	-426.969	-426.947	-426.848	-427.355	-427.493
VACUUM	-424.768	-426.816	-427.216	-427.009	-427.040	-427.164	-426.839	-426.818	-426.718	-427.226	-427.363

E start											
	HF	B2PLYP	B3LYP	M11	M06-2X	M06L	MN12L	mPW2PLY	PBE0	TPSSH	VSXC
H2O	-417.545	-419.129	-419.935	-419.738	-419.766	-419.873	-419.538	-419.166	-419.463	-419.946	-420.066
VACUUM	-417.529	-419.114	-419.921	-419.723	-419.753	-419.860	-419.524	-419.151	-419.450	-419.932	-420.053

G start											
	HF	B2PLYP	B3LYP	M11	M06-2X	M06L	MN12L	mPW2PLY	PBE0	TPSSH	VSXC
H2O	-417.461	-419.479	-419.860	-419.663	-419.690	-419.797	-419.461	-419.480	-419.387	-419.871	-419.993
VACUUM	-417.444	-419.465	-419.846	-419.648	-419.676	-419.785	-419.448	-419.466	-419.373	-419.857	-419.980

E vrtO											
	HF	B2PLYP	B3LYP	M11	M06-2X	M06L	MN12L	mPW2PLY	PBE0	TPSSH	VSXC
H2O					-419.469						
VACUUM					-419.358						

E vrtR											
	HF	B2PLYP	B3LYP	M11	M06-2X	M06L	MN12L	mPW2PLY	PBE0	TPSSH	VSXC
H2O					-419.800						
VACUUM					-419.732						

- VP

E Li_red

	HF	B2PLYP	B3LYP	M11	M06-2X	M06L	MN12L	mPW2PLY	PBE0	TPSSH	VSXC
H2O	-332.601	-332.601	-333.353	-333.131	-333.198	-333.308	-333.035	-332.636	-332.930	-333.368	-333.507
VACUUM	-332.561	-332.561	-333.315	-333.093	-333.160	-333.266	-332.995	-332.596	-332.891	-333.330	-333.472

G Li_red

	HF	B2PLYP	B3LYP	M11	M06-2X	M06L	MN12L	mPW2PLY	PBE0	TPSSH	VSXC
H2O	-332.900	-332.900	-333.266	-333.044	-333.110	-333.222	-332.947	-332.900	-332.841	-333.281	-333.421
VACUUM	-332.859	-332.859	-333.227	-333.004	-333.070	-333.177	-332.906	-332.861	-332.802	-333.242	-333.384

E red

	HF	B2PLYP	B3LYP	M11	M06-2X	M06L	MN12L	mPW2PLY	PBE0	TPSSH	VSXC
H2O	-325.122	-325.122	-325.858	-325.645	-325.706	-325.802	-325.519	-325.157	-325.458	-325.873	-325.994
VACUUM	-325.038	-325.038	-325.774	-325.558	-325.621	-325.718	-325.435	-325.072	-325.373	-325.789	-325.910

G red

	HF	B2PLYP	B3LYP	M11	M06-2X	M06L	MN12L	mPW2PLY	PBE0	TPSSH	VSXC
H2O	-325.423	-325.423	-325.772	-325.559	-325.620	-325.716	-325.432	-325.425	-325.371	-325.788	-325.910
VACUUM	-325.341	-325.341	-325.690	-325.473	-325.535	-325.634	-325.350	-325.342	-325.288	-325.705	-325.827

E Li_start

	HF	B2PLYP	B3LYP	M11	M06-2X	M06L	MN12L	mPW2PLY	PBE0	TPSSH	VSXC
H2O	-332.526	-332.526	-333.263	-333.041	-333.110	-333.221	-332.952	-332.560	-332.840	-333.280	-333.417
VACUUM	-332.405	-332.405	-333.142	-332.922	-332.990	-333.097	-332.830	-332.439	-332.719	-333.159	-333.300

G Li_start

	HF	B2PLYP	B3LYP	M11	M06-2X	M06L	MN12L	mPW2PLY	PBE0	TPSSH	VSXC
H2O	-332.813	-332.813	-333.171	-332.949	-333.016	-333.129	-332.858	-332.814	-332.746	-333.188	-333.326
VACUUM	-332.692	-332.692	-333.050	-332.830	-332.897	-333.005	-332.737	-332.693	-332.626	-333.068	-333.208

E start

	HF	B2PLYP	B3LYP	M11	M06-2X	M06L	MN12L	mPW2PLY	PBE0	TPSSH	VSXC
H2O	-325.060	-325.060	-325.780	-325.568	-325.631	-325.726	-325.447	-325.094	-325.380	-325.797	-325.918
VACUUM	-325.054	-325.054	-325.774	-325.561	-325.625	-325.721	-325.442	-325.088	-325.374	-325.791	-325.913

G start

	HF	B2PLYP	B3LYP	M11	M06-2X	M06L	MN12L	mPW2PLY	PBE0	TPSSH	VSXC
H2O	-325.349	-325.349	-325.690	-325.478	-325.539	-325.635	-325.355	-325.351	-325.289	-325.707	-325.829
VACUUM	-325.344	-325.344	-325.684	-325.471	-325.533	-325.630	-325.350	-325.345	-325.283	-325.701	-325.824

E vrtO

	HF	B2PLYP	B3LYP	M11	M06-2X	M06L	MN12L	mPW2PLY	PBE0	TPSSH	VSXC
H2O					-325.375						
VACUUM					-325.299						

E vrtR

	HF	B2PLYP	B3LYP	M11	M06-2X	M06L	MN12L	mPW2PLY	PBE0	TPSSH	VSXC
H2O					-325.699						
VACUUM					-325.613						

Schematic geometries of the Li^{+}- additive complexes as obtained from geometry optimization using C-PCM M06-2X/6-311++G(d,p)

- BOB

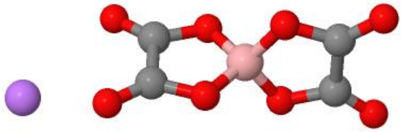

- DTD

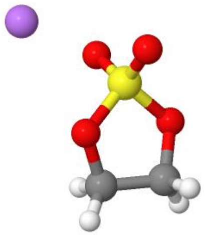

- EC

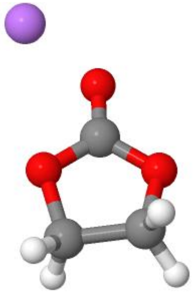

- ES

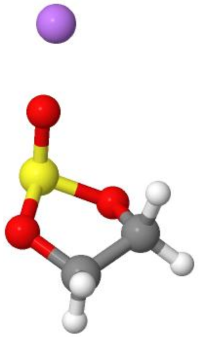

- FEC

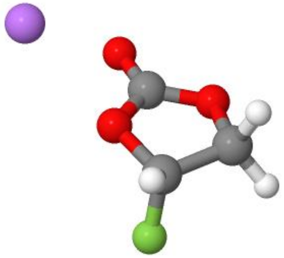

- PMC

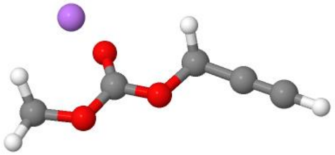

- VA

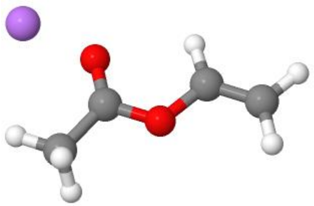

- VC

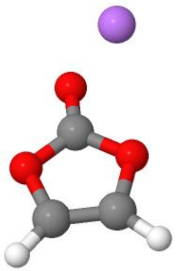

- VEC

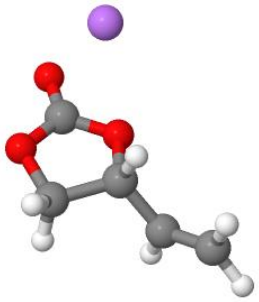

- VP

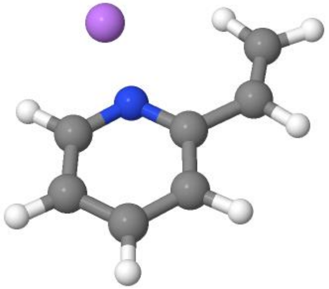

Supplement: Supplementary file 1 — (PDF 336 kb) [file 894_2016_3180_MOESM1_ESM.pdf]
